# Underwater Object Segmentation Based on Optical Features

**DOI:** 10.3390/s18010196

**Published:** 2018-01-12

**Authors:** Zhe Chen, Zhen Zhang, Yang Bu, Fengzhao Dai, Tanghuai Fan, Huibin Wang

**Affiliations:** 1 College of Computer and Information, Hohai University, Nanjing 211100, China; chenzhe@hhu.edu.cn (Z.C.); zz_hhuc@hhu.edu.cn (Z.Z.); 2 Laboratory of Information Optics and Opto-Electronic Technology, Shanghai Institute of Optics and Fine Mechanics, Shanghai 201800, China; buyang@siom.ac.cn (Y.B.); fzdai@siom.ac.cn (F.D.); 3 School of Information Engineering, Nanchang Institute of Technology, Nanchang 330099, China; fantanghuai@nit.edu.cn

**Keywords:** underwater object segmentation, optical features, level-set-based object segmentation, artificial light guidance

## Abstract

Underwater optical environments are seriously affected by various optical inputs, such as artificial light, sky light, and ambient scattered light. The latter two can block underwater object segmentation tasks, since they inhibit the emergence of objects of interest and distort image information, while artificial light can contribute to segmentation. Artificial light often focuses on the object of interest, and, therefore, we can initially identify the region of target objects if the collimation of artificial light is recognized. Based on this concept, we propose an optical feature extraction, calculation, and decision method to identify the collimated region of artificial light as a candidate object region. Then, the second phase employs a level set method to segment the objects of interest within the candidate region. This two-phase structure largely removes background noise and highlights the outline of underwater objects. We test the performance of the method with diverse underwater datasets, demonstrating that it outperforms previous methods.

## 1. Introduction

In recent years, interest in underwater computer vision has increased, taking advantage of the development of artificial illumination technologies and high-quality sensors [1,2,3,4]. To an extent, this progress has addressed some challenges for close-range imaging. However, for common underwater vision tasks where sight distance is large, extremely strong attenuation can seriously reduce the performance of artificial illumination. This, combined with strong hazing and interference from sky light, make underwater objects indistinguishable [5,6,7]. In such cases, commonly used image features, such as color, intensity and contours, are not well characterized [8].

Two key aspects to consider for underwater artificial light are optical characteristics such as its strength and spectrum, and its collimation, which is the region covered by the light. The former is accounted for by many underwater imaging and image processing methods, such as those extending underwater visibility [9,10]. However, no existing methods determine the importance of light collimation for underwater computer vision tasks.

The collimation of artificial light can provide novel cues to aid underwater object segmentation, since artificial light often collimates directly on objects of interest. Hence, we can initially identify candidate object regions following the guidance provided by artificial light. However, besides artificial light, sky light entering the camera can make local optical environments significantly inhomogeneous. Moreover, artificial light commonly mixes with other light, such as ambient light and object radiation. These factors tend to obstruct artificial light recognition.

In order to address these challenges, this paper investigates the potential of an optical feature-based artificial light recognition method in underwater environments. The recognition results are regarded as candidates for further object segmentation. The primary novelty of our method is that it is the first investigation that uses artificial light collimation as a cue for underwater object segmentation. The approach includes optical feature extraction and a calculation technique to recognize the region of artificial light, and artificial light recognition to guide underwater object segmentation.

The remainder of this paper is organized as follows. Section 2 reviews related work on underwater object segmentation and detection. Section 3 describes the optical feature extraction and calculation method for artificial light component recognition, which is used as a guidance in segmenting the candidate region. In Section 4, the level-set-based object segmentation method is performed within candidate regions, generating final underwater object segmentation results. Section 5 presents the results obtained by the proposed artificial light recognition and underwater object segmentation methods, and compares the performance of the proposed underwater object segmentation method with state-of-the-art methods using diverse underwater images. Finally, the conclusions are presented in Section 6.

## 2. Related Works

In contrast to the significant progress in ground image object segmentation, very few methods have been proposed to successfully segment objects in underwater data. In general, underwater object segmentation can be classified into two categories: methods based on prior knowledge of any special objects, and methods that are independent of any prior experience and are used for uncertain objects.

### 2.1. Prior-Knowledge-Based Methods

The advantage of prior-knowledge-based underwater object segmentation methods lies in their robust ability to identify special objects. For example, Yu et al. used a pre-defined color feature to detect and segment the artificial underwater landmarks (AULs) in an underwater vision system. This method has proven to be reliable for navigation when the camera is located near underwater objects [11]. Lee et al. designed a docking system using a LED ring with five large lights. Aiming to accurately track the position of an autonomous underwater vehicle, the structure of the docking-marks was carefully pre-designed and their brightness carefully adjusted. This information was input to a camera system as prior knowledge for performing detection of the docking marks and subsequent segmentation [12]. Besides the features given by these predesigned man-made objects, many types of prior knowledge can be introduced via machine learning methods. A typical sample is the use of Markov random fields in vision control systems [13,14]. In this method, the MRF model was used as a preprocessor to calculate the relationship between raw image data and corresponding corrected images, and the output of model was applied for detecting underwater coral objects. Additionally, many methods demonstrate the advantage of prior structural features for underwater object segmentation. Negre et al. presented a scale and rotationally invariant feature for self-similar landmark (SSL) detection. From the results, it was evident that structural features were more robust for underwater object detection than color features [15]. Maire et al. extracted rectangular Haar wavelet features and the rotation of integral images to detect uniquely identifiable objects for automatic docking control [16].

### 2.2. Methods without Prior Information

In contrast to prior-knowledge-based approaches, the second type of underwater object segmentation methods is independent of prior experience. This type of technique generally focuses on uncertain aquatic objects using a two-phase structure to overcome the challenge of variable underwater optical environments. For these systems, a preprocessor is commonly introduced to perform image enhancement and as a second stage for object segmentation. In principle, this strategy can better adapt to underwater environments. Lee et al. proposed an updated underwater image restoration method to correct the object colors. The contribution of image preprocessing to the underwater object detection was demonstrated by comparing the results before and after image preprocessing [17]. Kim et al. fused color correction and template matching to segment well-designed landmarks, and its performance was demonstrated by both indoor test water and outdoor natural pools [18]. Moreover, the two-phase structure in some studies includes a coarse-to-fine strategy. For example, Edgington et al. employed the Itti saliency detection method to first identify candidates. However, the usage of an Itti model in underwater environments is somewhat questionable. Features such as intensity, color, and orientation used by the Itti model are nominally suited for the typical ground scenes, while these features are unreliable in underwater conditions [19]. Further, the saliency detection method employed by Rizzini as a preprocessor can initially identify the candidate region of the objects. Object segmentation in the successive phase was achieved by using a low-pass filter [20]. Chuang et al. used the phase Fourier transform (PFT)-based saliency detection method to detect patches of underwater objects. The texture features were then extracted to classify fish images [21]. Zhu et al. integrated mean-shift oversegmentation and saliency detection methods to completely segment the underwater object. Image restoration was also used in this method for correcting underwater samples [22]. Recently, Chen et al. proposed a novel depth-feature-based region of interest (ROI) detection method. The ROI detection result is further corrected using Otsu’s method [23] for underwater object detection. From the presented results, the depth information was key for ROI detection; however, the method was vulnerable in environments where artificial and sky light seriously affect depth estimation [24]. Differences between the previously proposed work [24] and the method presented in this paper can be concluded in three aspects. Firstly, the previous underwater object detection method was achieved by a multi-feature fusion strategy and independent of any prior about underwater environments. In contrast, the method in this paper is totally based on a novel optical discipline given by the knowledge of underwater imaging: artificial light commonly collimates the underwater ROI (region of interest), thus can guide underwater object segmentation process. Secondly, the depth information in previous work was used to identify the ROI, which can only be achieved in relatively homogeneous environments. However, the underwater artificial light makes underwater optical environments inhomogeneous, which will block the usage of the previous method. In contrast, the method proposed in this paper is adaptive to inhomogeneous environments and has the ability to segment underwater objects using the cue originated from the artificial light. At last, the level set model is introduced in this paper to generate accurate underwater object segmentation results. However, the level set model is not taken advantage in the previous work [24], since the previous work aims to detect the existence of underwater objects. Hence, a simple OTSU model is enough and employed in the previous work.

### 2.3. Comparison to Previous Works

The work described above shows the success of coarse-to-fine strategies for underwater object segmentation and is an important motivation for research presented in this paper. However, in contrast to previously proposed methods, our approach is not specialized for any special objects and is independent of prior knowledge. Moreover, the novel cue provided by artificial light guidance is considered in our first step rather than the commonly used image enhancement or saliency detection methods.

### 2.4. Proposed Method

**Figure 1:**
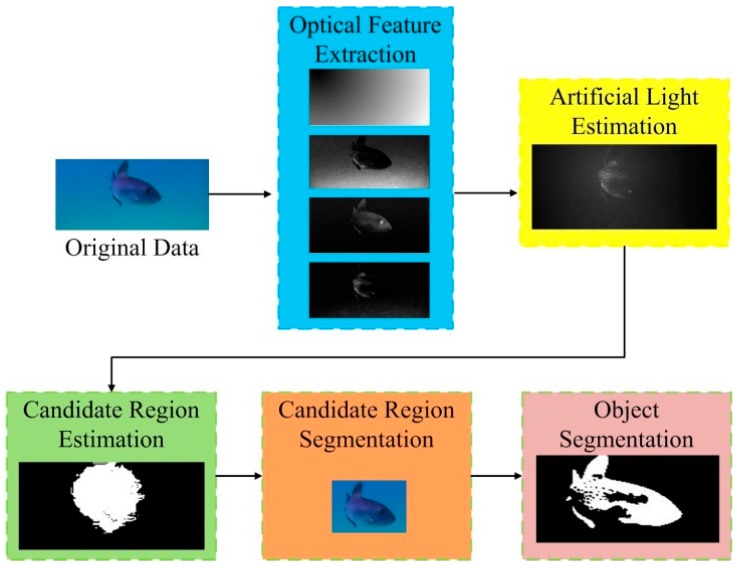
Framework of the proposed underwater object segmentation method.

The framework of the proposed method is shown in Figure 1. In the first phase, various optical features including the global intensity contrast, channel variation, intensity-position, and red channel contrast are extracted from the underwater images. These optical features are then calculated in a discrimination model to recognize the distribution of artificial light, which then guides the candidate region segmentation in the fourth stage, which is achieved by the simple OTSU method. Finally, a level-set method is applied to the candidate region, generating the final results for underwater object segmentation.

## 3. Optical Feature Extraction and Artificial Light Recognition

Artificial light can provide a novel cue for underwater object segmentation; however, its recognition using underwater optical features is a core issue. Optically, artificial light propagating through water presents many characteristics different from other light. The optical attenuation and light scattering as we know are two main factors which affect underwater light [25,26,27,28,29,30], as follows:

(i) *Optical attenuation*. The optical attenuation will cause a significant degeneration of underwater light power [25]. Besides, this attenuation effect exponentially changes with the sight distance. Since the distance between artificial lumination and reflectors, and the distance between reflectors and the camera are both limited, the degeneration of underwater artificial light is relatively small comparing to underwater natural light [26]. This will generate a higher intensity in local regions which at the same time presents a significant contrast to the surrounding regions. Moreover, the underwater light attenuation factor is also wavelength-selective [26]. For natural light in water, the red channel is extremely weak, which can be partly compensated by artificial light [27,28]. Hence, the red channel intensity of underwater artificial light is relatively high and has a large contrast to the natural light component.
(ii) *Light scattering.* The underwater light scattering factor will cause a halo effect in the region collimated by the artificial light [29,30]. In this region, the intensity of any points is inversely proportional to their distances from the brightest point. However, this halo effect is not presented in other regions due to the homogeneousness of underwater natural light.

Based on these characteristics above, the discriminative features of the artificial light can be extracted into four aspects. First, artificial light is more intense than other optical inputs, and consequently, its average intensity in the artificial light region is higher in the entire image. Second, the propagation distance of artificial light is shorter than other optical inputs, making artificial light channel variation smaller and the channels of the reflected artificial light more balanced. Third, a halo effect appears surrounding the collimation point. The intensity distribution within the artificial light region is exponentially modified with a radius, holding an intensity-position mode. Finally, as compared to the natural optical components, the red channel of artificial light is significantly more intense, which can cause its emergence in the artificial light region in the red channel.

### 3.1. Underwater Optical Feature Extraction

In general, four types of optical features are extracted in this paper: the global intensity contrast, channel variation, intensity position, and red channel contrast. The absolute difference in the intensity values is calculated to scale the intensity contrast at points *x* and *y*. The global intensity contrast at point *x* is the summation of these point-to-point differences as follows:

(1) $$C_{x}^{i}=\sum_{\forall y\in N}C(I_{x}^{i},I_{y}^{i})=\sum_{\forall y\in N}\left\|I_{x}^{i}-I_{y}^{i}\right\|$$

where $C(I_{x}^{i},I_{y}^{i})$ is the absolute difference between points *x* and *y* in the intensity value, N denote the points within underwater images, and the super scrip *i* is the label of the intensity.

The channel variation feature is computed as the summation of the differences between the channel intensity and the average intensity, as follows:

(2) $$V_{x}^{c}=\sqrt{\sum V(I_{x}^{c},I_{x}^{i})}=\sqrt{{\left(I_{x}^{r}-I_{x}^{i}\right)}^{2}+{\left(I_{x}^{g}-I_{x}^{i}\right)}^{2}+{\left(I_{x}^{b}-I_{x}^{i}\right)}^{2}}$$

where $V(I_{x}^{c},I_{x}^{i})$ is the variance of the point *x* in the r∗g∗b color space.

The intensity-position relation is scaled by the distance between point *x* and the point which has the largest intensity in the whole image, as follows:

(3) $$D_{x}^{d}=\exp\left(D(x,m)\right)=\exp\left(\sqrt{{\left(\xi_{1}-\xi_{2}\right)}^{2}+{\left(\gamma_{1}-\gamma_{2}\right)}^{2}}\right)$$

where D(x,m) is the Euclidean distance between point x and point m which has the largest intensity in the whole image, $x=\left[\xi_{1},\gamma_{1}\right]$, $m=\left[\xi_{2},\gamma_{2}\right]$ are the spatial coordinates of the points x and m.

The red channel contrast at point *x* is the summation of point-to-point differences in the red channel, as follows:

(4) $$C_{x}^{r}=\sum_{\forall y\in N}C(I_{x}^{r},I_{y}^{r})=\sum_{\forall y\in N}\left\|I_{x}^{r}-I_{y}^{r}\right\|$$

where $C(I_{x}^{r},I_{y}^{r})$ is the absolute difference between points *x* and *y* in the red channel, and the super scrip *r* is the label of the red channel.

**Figure 2:**
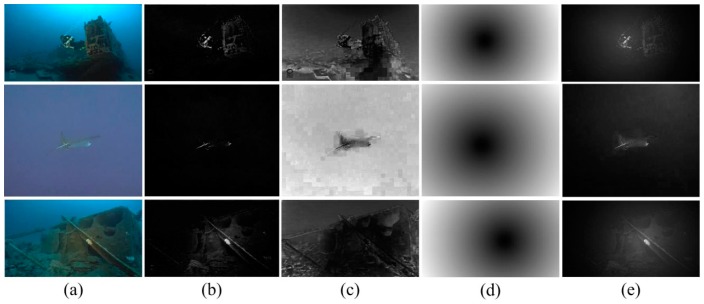
Underwater optical features. (**a**) original underwater image; (**b**) global intensity contrast; (**c**) channel variation; (**d**) intensity-position; (**e**) red channel contrast.

The optical feature extraction results are shown in Figure 2.

### 3.2. Artificial Light Recognition

Artificial light recognition follows four optical principles which are given or concluded by prior knowledge about underwater optical characteristics [25,26,27,28,29,30], as follows:

1. The inverse relationship between the global intensity contrast and the intensity position relation features: in the artificial light region, higher-contrast points are closer to the largest intensity point and vice versa.
2. The correspondence between the global intensity contrast and the red channel contrast features: within the artificial light region, an increase in the global intensity contrast corresponds to an increasing red channel contrast.
3. The inverse relationship between the global intensity contrast and the channel variation features: points with larger global intensity contrast have lower channel variation in the artificial light region, and vice versa.
4. The correspondence between the intensity-position relation and the channel variation features: within the artificial light region, the lower channel variation must be located in points that are closer to the maximum intensity point.

In this section, the discrimination function is proposed following the simulation of principles 1–4. Here, we generally use two-dimensional correlation [31] to scale the inverse relationship and correspondence between different optical features. According to the principle 1, the inverse relationship between the global intensity contrast and the intensity position relation can be established as $corr2(C_{}^{i},(1-D_{}^{d}))$, while according to the principle 2, the correspondence between the global intensity contrast and the red channel contrast can be calculated as $corr2(C_{}^{i},C_{}^{r})$. Similarly, the inverse relationship between the global intensity contrast and the channel variation (principle 3), and the correspondence between the intensity-position relation and the channel variation features (principle 4) can be calculated as $corr2(C_{}^{i},(1-V_{}^{c}))$ and $corr2(D_{}^{d},V_{}^{c})$, respectively. Large values of two-dimensional correlations will be obtained with significantly strong inverse relationship and correspondence between optical features. If all the two-dimensional correlations are of large values, the artificial light collimation is recognized in corresponding regions. Hence, the comprehensive discrimination function is formulated as the multiplicative of the correlation calculations as

(5) $$S=corr2(C_{}^{i},(1-D_{}^{d}))\times corr2(C_{}^{i},C_{}^{r})\times corr2(C_{}^{i},(1-V_{}^{c}))\times corr2(D_{}^{d},V_{}^{c})$$

where S is the discrimination function for the artificial light region; $C_{}^{i}$, $D_{}^{d}$, $C_{}^{r}$, and $V_{}^{c}$ are the matrices for the $C_{x}^{i}$, $D_{x}^{d}$, $C_{x}^{r}$, and $V_{x}^{c}$, respectively; and corr2( ) is used to calculate the two-dimensional correlation between matrices. According to Equation (5), if and only if all correlations (including inverse relationship and correspondence) reach significant high values, S will be of a high value which identifies the region covered by the artificial light. However, the divergence between correlations will result in a relative low value of S. In this case, the high intensity in local regions or the red channel may be caused by the sky light or bioluminescence which cannot correctly guide the underwater object segmentation. If all correlations are of extreme low values, the underwater environment may be seriously affected by any sources with specially designed spectrum.

We apply a threshold function to recognize the region of the artificial light as follows:

(6) $$L_{Atificial\;Light}=\left\{ \begin{array}{ll} 1 & if\;S>T\\ 0 & otherwise \end{array}\right.$$

where *T* is a threshold.

According to Equation (6), if $L_{Atificial\;Light}=1$, the artificial light exists, and the corresponding strength of the artificial light is calculated as

(7) $$W=\left(C_{}^{i}+C_{}^{r}-D_{}^{d}-V_{}^{c}\right)\times L_{Atificial\;Light}$$

where *W* is the strength of the artificial light.

### 3.3. Candidate Region Identification

**Figure 3:**
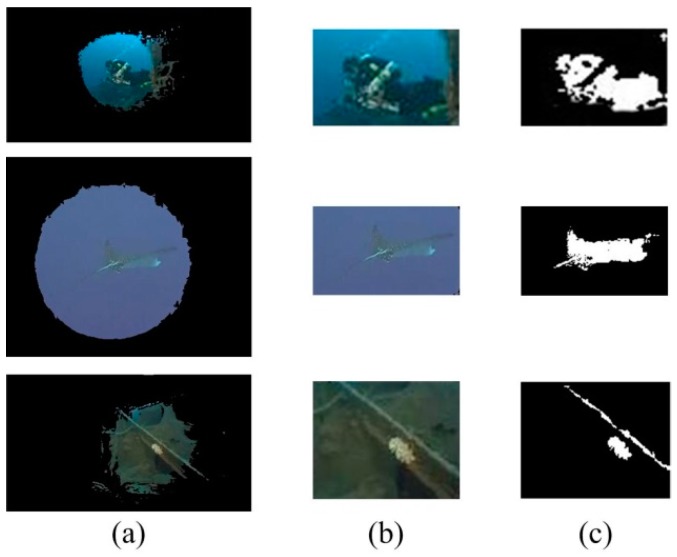
Underwater object candidate regions. (**a**) artificial light recognition; (**b**) candidate object region segmentation; (**c**) our object segmentation. The original images are shown in the first column of Figure 2.

Here, we use Otsu’s method to segment the map given by the artificial light recognition result *W*, extracting the candidate object regions [23]. The rationale behind the application of this method is twofold. Otsu’s method adapts well to processing the *W* maps, since the candidate region is distinguishable from the background in the gray histogram. However, Otsu’s method is efficient and linear with the size of the maps. Samples of segmentation results are shown in Figure 3a. Within the region given by Otsu’s method, the largest interior rectangle window is given to further identify the candidate regions, as shown in Figure 3b. In addition, Figure 3c shows the object segmentation result in the candidate regions. The contribution of the artificial light guidance can be demonstrated by Figure 3, that the artificial light region (Figure 3a) can correctly identify the candidate regions of underwater objects (Figure 2). Within the largest interior rectangle window of the artificial light region (Figure 3b), the object segmentation results (Figure 3c) can correctly occupy the ground truth and largely remove background noises.

## 4. Level Set Based Underwater Object Segmentation

In the post-processing phase, our underwater object segmentation results are generated by performing the level-set-based method in candidate regions. Specifically, we employ here a method using parametric kernel graph cuts [32]. Here, graph cuts that minimize the loss function in a kernel-introduced space are established as

(8) $$\psi_{\kappa }(\left\{u_{l}\right\},v)={\sum_{l\in L}\sum_{p\in R_{l}}\left(\phi (u_{l})-\phi (I_{p})\right)}^{2}+\alpha \sum_{\left\{p,q\right\}\in N}r(v(p),v(q))$$

where $\psi_{\kappa }(\left\{u_{l}\right\},v)$ is the measurement by the kernel-induced, non-Euclidean distances between the observations and the regions’ parameters, $u_{l}$ is the piecewise-constant model parameter of region, $I_{p}$ are the original image parameters, r(v(p),v(q)) is a smoothness regularization function established by the truncated squared absolute difference, and α is a positive factor.

**Figure 4:**
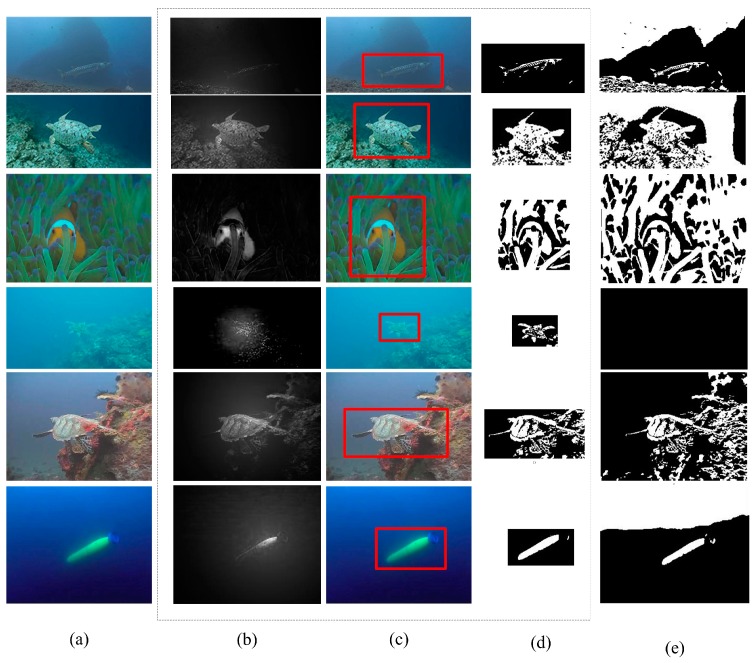
Phases of underwater object segmentation and comparison of results. (**a**) original underwater image; (**b**) artificial light recognition; (**c**) candidate object region segmentation; (**d**) our object segmentation; (**e**) object segmentation without artificial guidance.

This loss function is then minimized by an iterative two-stage method. In the first stage, the labeling is fixed and the loss function is optimized with respect to the parameter $\left\{u_{l}\right\}$. Then, an optimal labeling search is performed using the optimal parameter provided by the first stage. The level-set-based underwater object segmentation results with and without artificial light guidance are shown in Figure 4. The first column (Figure 4a) shows the original underwater images. The second column (Figure 4b) presents the artificial light recognition results, and the detected candidate regions are presented with a red rectangle in the third column (Figure 4c). The fourth column (Figure 4d) shows the results of the level-set-based method under the guidance of the artificial light, and in the last column (Figure 4e) the object segmentation results without artificial light guidance are presented. We find that the artificial light guidance significantly contributes to background removal, while without this prior guidance the segmented objects are indistinguishable and seriously overlap with background noise.

## 5. Experimental Results

### 5.1. Experiment Setup

The performance of the proposed method is demonstrated through comparison with five state-of-the-art saliency models. These models have shown excellent performance in various datasets and have been frequently cited in the literature. The compared methods include the updated saliency detection (HFT [33]) and statistical models (BGGMM [34], FRGMM [35]), and optimization (Kernel_GraphCuts [32], ROISEG [36]). Some of these methods have been successfully tested with underwater samples. Since our method is independent of training, new machine learning-based methods are not included in our experiment. The code for the baseline methods was downloaded from the websites provided by their authors.

### 5.2. Dataset

**Figure 5:**
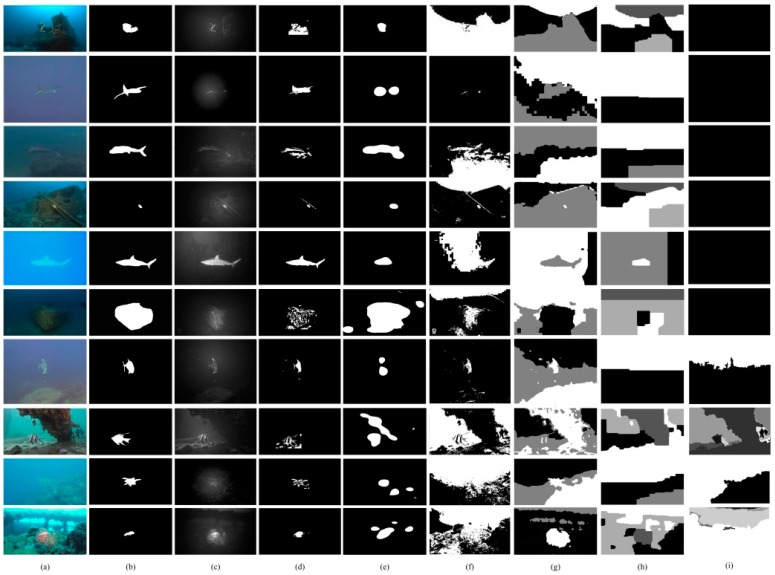
Result comparison. (**a**) underwater image; (**b**) ground-truth; (**c**) artificial light recognition; (**d**) our method; (**e**) HFT; (**f**) BGGMM; (**g**) FRGMM; (**h**) Kernel_GraphCuts; (**i**) ROISEG.

Underwater images available on YouTube were collected to establish the benchmark for the experimental evaluations [37,38,39,40,41,42,43,44,45]. These data were collected by marine biologists, tourists and autonomous underwater vehicles. More than 200 images were included in our underwater benchmarks. Some of these samples include participants located in coast areas where the underwater visibility is high while object appearances are seriously disturbed by large amount of optical noises [37,40,41,42,44] (i.e., the first, third, fourth eighth, ninth and the last rows in Figure 5). There are also samples presenting scenes under open seas where underwater objects are not clear due to the large sight distance [38,43,45] (i.e., the second, fifth and the seventh rows in Figure 5). Moreover, our dataset includes underwater images collected in environments with very weak natural light [39] (i.e., the sixth row in Figure 5). All test images were acquired by cameras with fixed focal length lens appended with underwater LED artificial illumination equipment. The highest brightness of these underwater lumination devices range from 5200 lm to 30,000 lm. The ground truth of each image was labeled by 10 experienced volunteers who major in researches of computer vision. Experimental samples used in this paper can be downloaded from our website [46]. The largest human visual contrast is used as the principle to segment underwater objects from background and the averaged results were deemed to be the ground truth. The images in this dataset differed in many aspects, including participants, context, and the optical inputs present. The diversity of these images ensures a comprehensive and fair evaluation of the methods. The threshold *T* for the decision function in Equation (6) is defined as 0.6 for all experiments.

### 5.3. Evaluation Methods

The PASCAL criterion [47] was used to evaluate the overlap of the detection results and ground truth

(9) $$C=\frac{\Omega^{\prime }\cap \Omega }{\Omega^{\prime }\cup \Omega }$$

where $\Omega^{\prime }$ is the detection; Ω is the labeled ground-truth; and *c* is degree of the overlap. Obviously, the larger the value of *c*, the more robust the algorithm. Moreover, the performance of our method was evaluated by six criteria [17], the precision (Pr), similarity (Sim), true positive rate (TPR), F-score (FS), false positive rate (FPR), and percentage of wrong classifications (PWC).

(10) $$\mathrm{Pr}=\frac{tp}{tp+ft},\;\mathrm{TPR}=\frac{tp}{tp+fn},\;\mathrm{Fs}\;=\;2\;\times \;\frac{\mathrm{Pr}\times \mathrm{TPR}}{\mathrm{Pr}+\mathrm{TPR}}\;\mathrm{Sim}=\frac{tp}{tp+fp+fn},\;\mathrm{FPR}=\frac{fp}{fp+tn},\;\mathrm{PWC}=100\times \frac{fn+fp}{tp+tn+fp+fn}$$

where *t_p_*, *t_n_*, *f_p_* and *f_n_* denote the numbers of the true positives, true negatives, false positives, and false negatives, respectively.

### 5.4. Results

Figure 5 shows the experimental results of our artificial light recognition, our object segmentation methods and five compared methods with ten typical test images. The optical environments of the test underwater image (Figure 5a) are diverse. The participants in the underwater images are different. Moreover, random image noise is obvious in these images. Together, these factors impose a serious challenge for object segmentation.

The first column in Figure 5 shows the original images; the second column is the ground-truth; the third and fourth columns respectively present the results of our artificial light recognition and our object segmentation methods. The last five columns respectively present the results given by the five compared methods. From the results, the most competitive performance is demonstrated by our method which can correctly segment underwater objects and remove the complicated background. The most comparable results are presented in the fifth column, provided by the HFT method. From the results, the HFT method works well in relatively pure scenes, such as the samples in the second and third rows. However, its performance is significantly poorer in scenes including textural backgrounds, such as the samples in the last three rows. From the results shown in the sixth and seventh columns, the statistical model can detect the objects of interest, but do not have the capability to recognize objects from the background. Moreover, the statistical models are also vulnerable to optical noise. As the result, the detected objects of interest in the fourth and fifth columns seriously overlapped with the background and the optical noise. Generally, without the guidance of the artificial light, the level-set-based method could not adapt to the underwater scenes, providing insignificant and vague results. Recall that the second phase of our proposed method is established by the level-set-based method (Kernel_GraphCuts), so that the importance of the artificial light guidance can be highlighted by comparing the results in the fourth and the eighth columns. The objective of these level-set-based methods is to segment the image into discrete regions that are homogeneous with respect to some image feature. However, for underwater images, hazing and ambient scattered light significantly inhibit the emergence of the objects of interest. Considering the entire image, the margin between the objects and background is relatively indistinguishable, while other transitions, such as those between sky and ambient parallel light, may be mistaken as regional boundaries. Alternatively, our method uses the artificial light collimation to guide the object segmentation process. The level-set-based image segmentation processing in our method focuses on the candidate regions that present a relatively higher contrast between objects and the surrounding background. In this case, the object contours are the most optimal boundaries with which to minimize the loss function (Equation (8)). This is the factor underlying the success for our underwater object segmentation method.

To further examine the quantitative performance of our method, Table 1 shows the differences in the average performance of the different methods on the 200 underwater images. The PASCAL criterion ($\bar{C}$), the precision (Pr), true positive rate (TPR), F-score (FS), similarity (Sim), false positive rate (FPR), and percentage of wrong classifications (PWC) are used here as the quantitative criteria. From the results, our method performed best, scoring the first in four criteria and the second in three criteria. Specifically, the highest scores of the criteria $\bar{C}$, Pr, TPR and Sim indicate that our segmentation results can exactly occupy bodies of underwater objects. The robustness against the background noises is demonstrated by the criteria FS, FPR and PWC. According to these three criteria, the best performance is achieved by the HFT method, which, however, mistakes parts of the objects as the background. As a result, there are many holes existing in the results given by the HFT method (Figure 5). Although the second rather than the first best performance is given by our method referred to the criteria FS, FPR and PWC, our method can remove most of background noises, demonstrating the contribution of our artificial light guidance for underwater object segmentation. Generally, the best comprehensive performance is achieved by our method. However, the other four compared methods (BGGMM, FRGMM, Kernel_GraphCuts and ROISEG), as shown in Table 1, scored worse than the proposed method. This indicates that these four methods cannot well adapt to the underwater environments and the results given by these methods are insignificant, which is in accordance with the qualitative results shown in Figure 5.

## 6. Conclusions

The work presented in this paper is a novel investigation of the usage of optical features for underwater computer vision. Various features, such as the global intensity contrast, channel variation, intensity position, and the red channel contrast, were extracted and jointly used to establish a decision function for artificial light recognition. The recognition results provide strong guidance for underwater object segmentation. This new method overcomes challenges caused by difficult underwater optical environments, such as light attenuation and hazing. The outstanding performance of our method was demonstrated by experiments on diverse underwater images.

Although the evaluation results presented in our paper preliminarily demonstrate the performance of the artificial light guidance for underwater object segmentation, the robustness of our method is required to be tested on more underwater images. Moreover, this work is a starting point that sheds light on the contribution of optical models for underwater computer vision tasks. In further work, we aim to introduce additional prior optical knowledge into state-of-the-art computer vision methods by model reconstruction, establishing more elaborate models for underwater optical environments.

## Figures and Tables

**Table 1:** Average performance comparison of HFT, BGGMM, FRGMM, Kernel_GraphCuts, ROISEG and our method using diverse underwater image data.

Method	$\bar{C}$	P_r_	TPR	FS	Sim	FPR	PWC
HFT + OTSU	0.5628	0.5426	0.7912	0.5858	0.4352	0.0331	3.5898
BGGMM	0.3075	0.3076	0.7480	0.2987	0.2071	0.2329	23.3065
FRGMM	0.3708	0.3387	0.8475	0.4109	0.3150	0.1253	12.5406
Kernel_GraphCuts	0.1178	0.2345	0.7198	0.1782	0.1156	0.2259	23.9979
ROISEG	0.1042	0.3509	0.2804	0.0906	0.0656	0.1219	11.8211
Our method	0.7164	0.6327	0.7968	0.5162	0.4479	0.0355	7.1233
